# The role of memory T cells in *Echinococcus granulosus*‐induced sensitization

**DOI:** 10.1002/iid3.948

**Published:** 2023-08-10

**Authors:** Jing‐Ru Zhou, Xiao‐Xuan Du, Xianyidan Abulajiang, Wuer Geli, Xue‐Li Pu, Subi Tailaiti, Jia‐Ying Lin, Yu‐Qian Li, Jian‐Rong Ye

**Affiliations:** ^1^ Department of Anesthesiology The First Affiliated Hospital of Xinjiang Medical University Urumqi China; ^2^ Graduate School of Xinjiang Medical University Urumqi China; ^3^ Department of Anesthesiology The Sixth Affiliated Hospital of Xinjiang Medical University Urumqi China

**Keywords:** allergic reaction, BALB/c mice, *Echinococcus granulosus*, IL‐23, memory T cells

## Abstract

**Objective:**

To investigate the changes in memory T cells and the related factors in mice by the establishment of a BALB/c mouse model of *Echinococcus granulosus*‐induced sensitization.

**Methods:**

A sensitized BALB/c mouse model was established by intraperitoneal injection of *E. granulosus*. A control group (CTRL), a nonsensitized group infected with *E. granulosus* (CE), and a sensitized group infected with *E. granulosus* (ANPC) were set up. The pathological changes in lung tissue in mice, the change in memory T cells (CD4 Tm), and the change in peripheral blood nucleated interleukin‐23 (IL‐23) were detected using HE staining, flow cytometry, and liquid‐phase multiple protein quantification techniques, respectively.

**Results:**

The individual percentage of mouse memory T cells was 9.14 ± 0.45, 25.23 ± 0.17, and 13.29 ± 0.32 in the CTRL, CE, and ANPC groups, respectively. The percentage of memory T cells in the ANPC group was higher than that in the CTRL group (*t* = 18.410, *p* < .001) but lower than that in the CE group (*t* = −80.147, *p* < .001). The levels of IL‐23 in peripheral blood of mice in the CTRL, CE, and ANPC groups were 225.76 ± 27.16, 359.21 ± 28.67, and 215.69 ± 22.69, respectively. The level of IL‐23 in peripheral blood of mice in the ANPC group was lower than that in the CE group (*t* = 9.609, *p* < .001), and there was no statistical difference with the CTRL group (*t* = 0.697, *p* = .502).

**Conclusion:**

In the BALB/c mouse model of *E. granulosus*‐induced sensitization, the expression of IL‐23 in peripheral blood increased, and the memory T cell proliferated and became activated; there was a decrease in the content of IL‐23 in peripheral blood and number of activated memory T cells in the sensitization group infected with *E. granulosus*. The *E. granulosus*‐induced allergic reaction was related to IL‐23 and the activation of memory T cells.

## Graphical abstract

In the BALB/c mouse model of *Echinococcus granulosus*‐induced sensitization, the expression of IL‐23 in peripheral blood increased, and the memory T cell proliferated and became activated; there was a decrease in the content of IL‐23 in peripheral blood and number of activated memory T cells in the sensitization group infected with *E. granulosus*. The *E. granulosus*‐induced allergic reaction was related to IL‐23 and the activation of memory T cells.

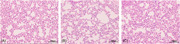

## 1 INTRODUCTION

Echinococcosis is a parasitic disease caused by infection with *Echinocococcus*. Echinococcosis can be divided into cystic echinococcosis (CE) and alveolar echinococcosis (AE), which are caused by infection with *Echinococcus granulosus* and *Echinococcosis multilocularis*, respectively.^1^, ^2^, ^3^ The disease is prevalent in pastoral regions globally. In China, patients with the disease are primarily found in the pastoral regions of China's northwest, including Xinjiang, Gansu, Ningxia, Qinghai, and Inner Mongolia. The population in China infected by echinococcosis has surpassed 66 million, resulting in an annual economic loss of about RMB 3 billion.^4^ Echinococcosis is a zoonotic disease, a number of herbivorous and omnivorous animals act as intermediate hosts of *Echinococcus*. They become infected by ingesting the parasite eggs in contaminated food and water, the eggs produce *E. granulosus* vesicles in the intermediate hosts.^5^, ^6^, ^7^ The most lethal complication of the condition is anaphylactic shock caused by the rupture of the cyst wall following a forceful external impact, which can result in the death of the host if not treated promptly and effectively.^8^, ^9^, ^10^ However, the mechanism by which CE induces anaphylaxis in intermediate host remains unknown. The establishment of the mouse model with cystic *E. granulosus*‐induced sensitization and the systematic in‐depth study on clinical *E. granulosus*‐induced anaphylactic shock and its pathogenic mechanism as well as the prevention and control theory can therefore provide a scientific basis for the prevention and treatment strategies for patients with anaphylactic shock caused by CE.

## 2 MATERIALS AND METHODS

### 2.1 Experimental animals

BALB/c female mice, 4 to 8‐week‐old, weighing 20 ± 2 g, SPF grade, were purchased from the Experimental Animal Center of the First Affiliated Hospital of Xinjiang Medical University.

### 2.2 Reagents and instruments

Hemolysin, IL‐23, and flow cytometry antibodies of CD4‐FITC, CD44‐PE, and CD62L‐PE‐CF59 were purchased from Becton, Dickinson and Company, USA. We used the DXflex flow cytometer (Beckman Coulter).

### 2.3 Experimental methods

#### 2.3.1 Preparation of protoscoleces of *E. granulosus* and cyst fluid

Fresh livers of sheep naturally infected with *E. granulosus* were collected from a slaughterhouse in Urumqi, Xinjiang, China. The contents of the noncalcified, noninfected, intact unicystic *E. granulosus* vesicles were aseptically extracted from the livers of sheep infected. The contents were transferred to sterile centrifuge tubes (CORNING), centrifuged at 1500*g* for 10 min to extract the supernatant, which was then treated with endotoxin removal, and filtered through a 0.22 μm membrane (Millipore) and quantified for protein concentration before being frozen at −80°C. To naturally eliminate fragments of the brood capsule from the protoscoleces sediment, it was digested with 1% pepsin (BioFroxx) and then rinsed three times with sterile PBS containing 100 U/mL penicillin‐streptomycin solution (Gibco). The sediment was stained with 0.5% eosin (BKMAMLAB) for 5 min, and the number of active protoscoleces (>90%) was counted microscopically and diluted to 10,000 protoplasts/mL in suspension with sterile PBS containing penicillin‐streptomycin solution and kept on standby.^11^

#### 2.3.2 Grouping and modeling of animals

Eighteen mice were randomly divided into three groups: a normal group, a nonsensitized group, and a sensitized group. The mice in the normal group were injected intraperitoneally with saline (0.1 mL/10 g); the mice in the nonsensitized group were injected intraperitoneally with 10,000 protocephalic larvae/mL at 0.2 mL each mouse, and 90 days later with saline (0.1 mL/10 g); the mice in the sensitized group were injected intraperitoneally with 10,000 protocephalic larvae/mL at 0.2 mL each mouse, and 90 days later with crude cyst solution (0.1 mL/10 g) to be sensitized. One hour following the final injection, all mice in the three groups were injected and anesthetized with propofol (Sichuan Guorui Pharmaceutical Company Limited), and euthanized using the cervical dislocation method after angular vein blood sampling.

#### 2.3.3 Detection of pathological changes in lung tissue with HE staining

Immunohistochemistry was used to examine the expression of histamine in the lung tissues of mice. The lung tissues were embedded in paraffin wax, sectioned into 5‐μm‐thick sections, then spread out and baked to dry. The dried sections were dewaxed with xylene before being rehydrated with an ethanol gradient (100% to 70%). The hydrated sections were stained with hematoxylin solution for 5 min, followed by decolorization with 1% acidic ethanol (1% HCl, 70% ethanol) five times, and then rinsed with distilled water to remove any excess acidic alcohol. The sections were stained for 3 min with eosin staining solution, dehydrated with graded alcohol, and made transparent using xylene. Finally, the sections were microscopically (Leica) detected and photographed.

#### 2.3.4 Detection of memory T cells with flow cytometry

Blood was drawn from the angular vein of the mice using an EDTA vacuum anticoagulant sampling vessel (SANLI). For the detection of memory T cells, 100 μL of anticoagulant whole blood and CD4/CD44/CD62L antibody were added to each sample tube and cultured for 30 min at room temperature in the dark; 10× red blood cell lysate (Solarbio) was measured and diluted with double distilled water by volume at a 1:9 ratio. Per 100 μL of whole blood was add with 2 mL of 1× red blood cell lysate and lysed at room temperature for 12 min until the cell suspension was clear and transparent. The supernatant was then discarded after centrifugation at 1500*g* for 5 min, and the cells were washed three times with PBS; 500 uL of PBS washing liquor was added to each tube, and the contents were analyzed with the DXflex flow cytometer.

#### 2.3.5 The measurement of IL‐23 in mouse peripheral blood using the liquid‐phase multiplex protein quantification technique (CBA)

(1) Preparation of standard product: The standard microspheres were poured into a 15 mL centrifuge tube diluted with 4 mL of sample diluent, mixed gently, and let to stand for 15 min.
(2) Preparation of microspheres: The total amount of captured microspheres to be diluted for the experiment was determined. Each reaction required 50 µL of diluted captured microspheres. After determining the required quantity of captured microsphere and the volume of microsphere dilution, the captured microspheres were evenly mixed and transferred to fresh tubes.
(3) Preparation of antibody: 50 µL of diluted antibody was required for each reaction. After determining the required amount of each antibody and the volume of antibody diluent, the mixed antibodies were transferred to a fresh tube.
(4) Assay: The 50 µL mixed microspheres were added to each tube. In each tube, 50 µL of the corresponding standard product or sample was added, mixed evenly, and cultured at room temperature for 1 h. Then, 50 µL of the mixed antibodies were added to each tube, mixed gently, and cultured at room temperature for 1 h. Then, 1.0 mL of wash buffer was added to the cultured solutions, centrifuged at 300*g* for 5 min, and the supernatant was discarded. Finally, 300 µL wash buffer was added to the sediment to resuspend the microspheres for analysis with flow cytometry.

### 2.4 Data analysis

All data were statistically analyzed using SPSS 23.0 software, and the measurement data are expressed as mean ± standard deviation ($\bar{x}\pm s$) The acquired data were analyzed using one‐way ANOVA. Flow data were plotted using FlowJo analysis software.

## 3 RESULTS

### 3.1 HE staining

**Figure 1:**
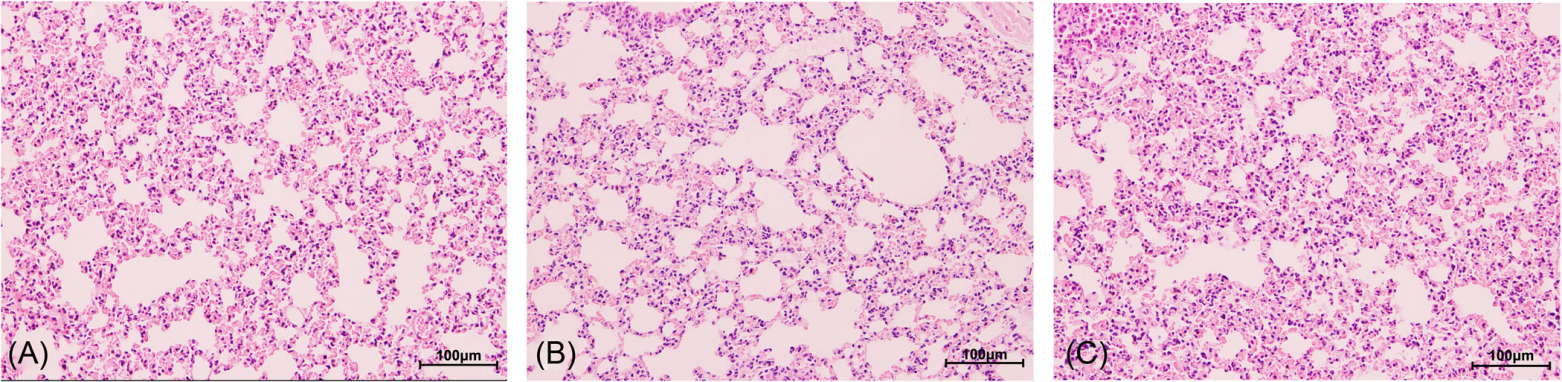
HE staining results of lung tissue in mice sensitized and unsensitized by hydatid cyst fluid. (A) Control group; (B) Nonsensitized group infected with *Echinococcus granulosus*; (C) Sensitized group infected with *E. granulosus*. The magnification used is ×200.

The lung tissues of mice in the control group contained intact bronchial epithelium and no infiltration of inflammatory cells; the lung tissues of mice in the sensitized group showed obvious inflammatory cell infiltration around fine and small bronchial walls and accompanying blood vessels, with the inflammatory cells including lymphocytes, eosinophils, and neutrophils, indicating that the anaphylactic reaction model induced by *E. granulosus*‐infected mice attacked by the crude cyst fluid from sheep infected with *E. granulosus* was successfully constructed (see Figure 1).

### 3.2 Effect of *E. granulosus* cyst fluid sensitization on changes in memory T cells of mice

**Figure 2:**
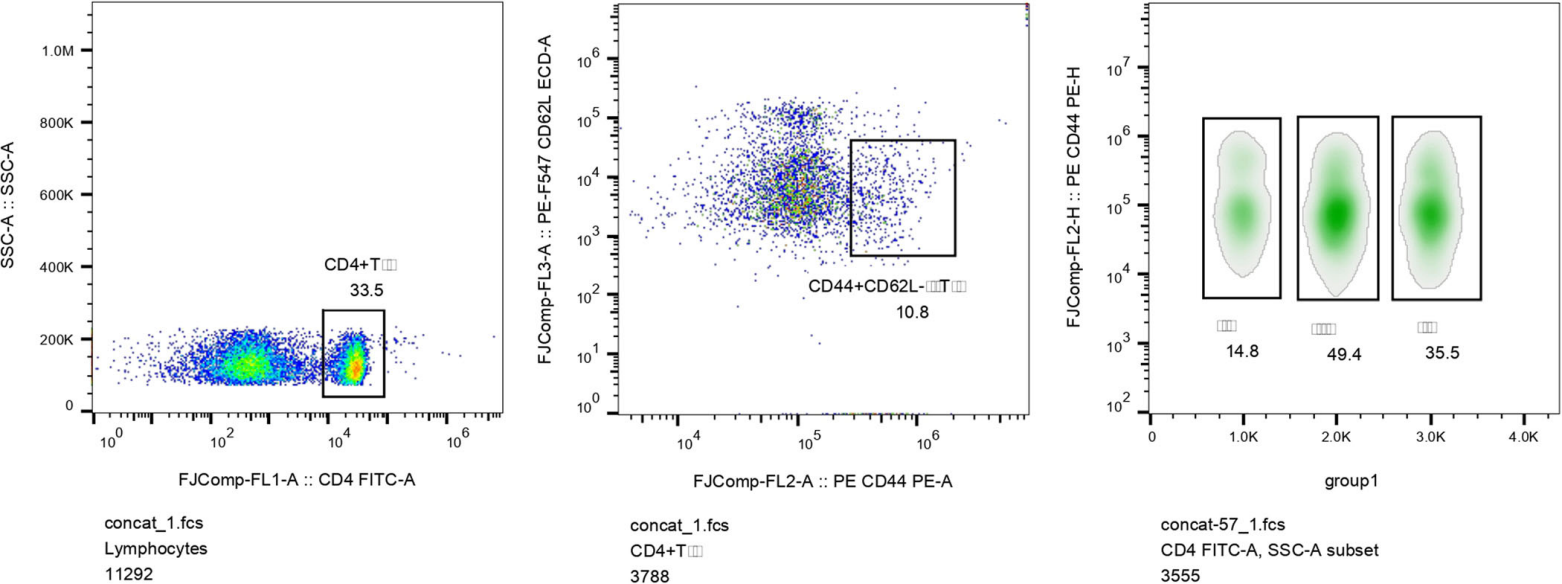
Results of memory T cells in BALB/c mice sensitized by hydatid cyst fluid by flow cytometry.

After *E. granulosus* cyst fluid‐induced sensitization, alterations in the memory T cells of mice in each subgroup were observed by flow cytometry. Compared with the control group, the percentage of memory T cells of mice in the nonsensitized group increased by 176% (*t* = 81.93, *p* < .001); the percentage of memory T cells of mice in the sensitized group increased by 45.4% (*t* = 18.410, *p* < .001). The percentage of memory T cells of mice in the sensitized group decreased by 47.32%, compared to the nonsensitized group (*t* = −80.741, *p* < .01). The results are shown in Table 1 and Figure 2.

**Table 1:** The influence of hydatid fluid on DC percentage in BALB/c mice.

Group	Percentage of memory T cells	*n*
Control group	9.14 ± 0.45	6
Nonsensitized group	25.23 ± 0.17*	6
Sensitized group	13.29 ± 0.32** ^,^ ***	6

Abbreviation: DC, dendritic cell.

* Compared with the control group: *p* < .001.

** Compared with the control group: *p* < .01.

*** Compared with nonsensitized group: *p* < .01.

### 3.3 Effect of *E. granulosus* cyst fluid sensitization on changes in cytokine IL‐23 in peripheral blood of mice

**Figure 3:**
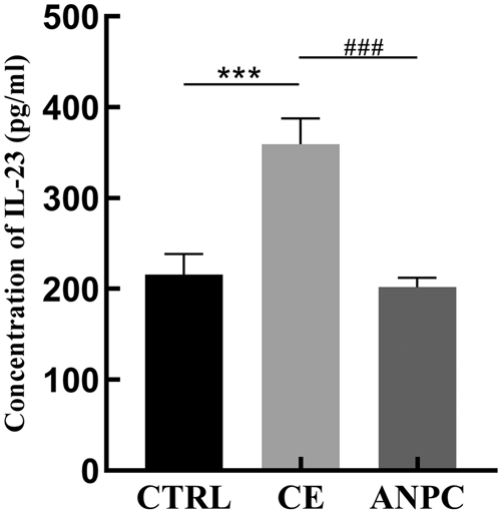
The effect of hydatid cyst fluid sensitization on the changes of IL‐23 in peripheral blood of BALB/c mice was detected by liquid‐phase multiple protein quantification technique. *compared with the control group, ***p < .001; #compared with nonsensitized group, ^###^ *p* < .001.

Using a liquid‐phase multiplex protein quantification approach, the variations in cytokine IL‐23 in the peripheral blood of mice in each group following sensitization induced by *E. granulosus* cyst solution were assessed (see Table 2 and Figure 3). The level of IL‐23 in peripheral blood was substantially lower in the sensitized group than in the nonsensitized group (*t* = 9.609, *p* < .001), but there was no statistically significant difference between the sensitized group and the control group. (*t* = 0.697, *p* = .502). IL‐23 levels in peripheral blood were significantly higher in the nonsensitized group than in the control group (*t* = −8.277, *p* < .001).

**Table 2:** Difference of IL‐23 changes in peripheral blood cytokines of BALB/c mice after sensitization with cyst fluid.

Group	Peripheral blood cytokine IL‐23	*n*
Control group	225.76 ± 27.16	6
Nonsensitized group	359.21 ± 28.67*	6
Sensitized group	215.69 ± 22.69**	6

* Compared with the control group: *p* < .001.

** compared with nonsensitized group: *p* < .001.

## 4 DISCUSSION

For hosts with CE caused by *E. granulosus* infection, the rupture of vesicles and the exudation of cyst fluid caused by various factors such as surgery, trauma, and spontaneous action, can result in anaphylactic shock, with severe consequences and even death.^12^ Anaphylactic shock caused by CE is a special type of allergic reaction related to parasite infection and triggered by parasitic products such as cyst fluid.^13^ As a result of the unique immune condition of patients with CE, the clinical signs and immunological characteristics of patients with anaphylactic shock differ from those of patients with type I allergy when the cyst fluid is released owing to vesicle rupture. Li Yimei's team conducted a retrospective study on 446 cystic echinococcosis patients who received surgical treatment and concluded that the Anaphylaxis caused by CE may be caused by rapid onset combined with cytotoxic anaphylaxis.^14^ The symptoms of patients cannot be alleviated by adrenaline alone. From the perspective of antibodies and cytokines, echinococcus cyst fluid antigens and secretions can serve as allergens, which can cause the body to produce specific IgE and IgG1 antibodies, further leading to an increase in IL‐4 and IL‐10; The increase in IgG1 levels further exacerbates allergic reactions through antibody dependent cell mediated cytotoxicity.^15^, ^16^ Despite this, the pathogenesis of anaphylactic shock triggered by CE remains unknown. Consequently, many individuals have perished in the absence of prompt and appropriate diagnosis and treatment.

Memory T cells mainly consist of 2 subgroups: CD4^+^ memory T cells and CD8^+^ memory T cells. CD45RO^+^ is the surface marker of human memory T cells and CD45RA^+^ is the surface marker of initial T cells. Depending on the expression of homing receptors, central‐type and effector‐type memory T cells can be differentiated—CCR7^+^ and CD62L^+^ are central‐type and CCR7^−^ and CD62L^−^ are effector‐type.^17^ The surface markers of memory T cells in mice differ from those in humans, and the expression level of CD44 in mice is used to define memory T cells—cells with low expression of CD44 are defined as naive T cells and cells with high expression of CD44 are defined as memory T cells. CD62L is then used to distinguish central and effector memory T cells—cells with CD62L^+^ are defined as central‐type and cells with CD62L^−^ are defined as effector‐type.^18^ Since IL^−^23 mainly acts on effector memory T cells,^19^ the surface marker of CD44highCD62L^−^ was selected to detect the number of memory T cells in this study. According to the findings of this study, the proportion of memory T cells (CD4^+^ Tm) mice in the sensitized group was considerably higher than in the control and nonsensitized groups. Tm with CD4^+^ are a very significant cell subset that play a crucial role in a range of disorders, such as graft‐versus‐host disease, malignancies, metabolic diseases, and pathogenic infections, as well as in vaccinations.^20^ Moreover, CD4^+^ Tm is related to the field of allergic asthma, as the proliferation and functional activation of CD4^+^ Tm influence the release of a series of downstream Th2 cytokines and the cascade of inflammatory reactions. It is widely recognized that an unbalanced Th1/Th2 ratio is one of the key pathogenic processes behind the development of allergic responses. Hence, CD4^+^ Tm was the main target in this study.

IL‐23, a cytokine discovered in 2000, is a member of the IL‐6 family and consists of two subunits: p19 and p40; p40 is also a subunit of IL‐12. IL‐23 exerts its biological function by interacting with the IL‐23 receptor (IL‐23R) on the cell surface.^21^ Numerous cells express IL‐23R on their surface, including dendritic cells (DCs), macrophages, and polarized lymphocytes, and hence IL‐23 operates in a variety of cells and is associated with the progression of diseases such as tumors, infections, and autoimmune disorders.^22^ Some studies have shown that IL‐23 is necessary for type 17T helper cell (TH17 cells) to mediate autoimmune inflammation. In the absence of IL‐23, TH17 cells stagnate in their early development, thus unable to maintain the production of IL‐17 and up regulate IL‐17 receptor α Expression of Chain (IL‐17Ra).^23^ The IL‐23/IL‐17 inflammatory response axis plays a crucial role in a number of autoimmune diseases and inflammatory‐responsive diseases; a study on the mechanism of immune tolerance in hydatid disease of liver revealed that IL‐23 was involved in the immune tolerance hydatid disease and the inflammatory response.^24^

Treg cells and TH17 cells are two different subpopulations of Th1 and Th2, and they control inflammatory reaction in a balanced way. Wen team found that the number of Treg cells in peripheral blood of CE infected patients is higher than that of normal people, leading to the imbalance of Treg/Th17 cells, which may play an important role in the immune escape mechanism of fine‐grained Echinococcosis.^25^ IL‐23 has no direct effect on Treg cells, but its activation γδ T cells can relieve Treg cell responses.^26^ It has been reported that memory T cells express IL‐23R on their surface and that IL‐23 can boost memory T cell proliferation and cytokine release in normal organisms.^27^ The results of this study revealed that compared to the control group, the expression levels of memory T cells and IL‐23 were significantly higher in both the nonsensitized and sensitized groups, which indicates that after infecting the mice in the non‐sensitized group, *E. granulosus* achieved immune escape through the increased expression of IL‐23, which then promotes the proliferation and activation of memory T cells, resulting in the secretion of more Th1‐related cytokines, such as IFN‐γ, IL‐10, and IL‐17. Compared with the nonsensitized group, the levels of memory T cells and IL‐23 were significantly lower in the sensitized group indicating that the organism promotes the response to allergic reaction by downregulating the number of memory T cells and reducing the expression of IL‐23 after the sensitization induced by *E. granulosus* cyst fluid.

The purpose of this study is to investigate the role of memory T cells and IL‐23 in allergic reactions caused by cystic echinococcosis, but its specific mechanism of action has not been explored. Therefore, future studies can target the knockout of IL‐23 through gene deletion or silencing to further reveal the specific association between memory T cells and allergic reactions caused by cystic echinococcosis. In addition, due to the difficulty in collecting clinical specimens, this study was unable to collect specimens from clinical patients. Therefore, further elucidation of the allergic reactions caused by cystic echinococcosis in the human body requires the collection and accumulation of frontline clinical specimens.

## 5 CONCLUSION

In conclusion, in the *E. granulosus* model, upregulation of IL‐23 stimulates and promotes the proliferation of memory T cells, which in turn drives memory T cells to release Th1‐related factors resulting in immunological escape of *E. granulosus* and allergic reactions induced by the resistance to hydatid‐cyst fluid. The results of this study revealed that the imbalance between memory T cells and IL‐23 plays a significant role in the anaphylactic shock induced by hydatid‐cyst fluid. This work provides a significant scientific foundation for the prevention and management of anaphylactic shock in patients with hydatid disease.

## AUTHOR CONTRIBUTIONS

**Jing‐Ru Zhou**: Writing—original draft (lead); formal analysis (lead). **Xiao‐Xuan Du**: Investigation (lead); writing—review and editing (equal). **Xianyidan Abulajiang**: Data curation (lead); formal analysis (equal). **Wuer Geli**: Methodology (equal); resources (equal). **Xue‐Li Pu**: Methodology (equal). **Subi Tailaiti**: Resources (lead). **Jia‐Ying Lin**: Validation (lead). **Yu‐Qian Li**: Data curation (equal); formal analysis (equal). **Jian‐Rong Ye**: Conceptualization (lead); writing—review and editing (lead).

## CONFLICT OF INTEREST STATEMENT

The authors declare no conflict of interest.

## ETHICS STATEMENT

The related content of the experimental research program of this project has been earnestly discussed by the Laboratory Animal Use and Management Committee of the First Affiliated Hospital of Xinjiang Medical University (No.IACUC‐20200318‐04), and is considered to meet the ethical requirements and approved by vote.

## Data Availability

All data generated or analyzed during this study are included in this article. Further enquiries can be directed to the corresponding author.
